# Moral Injury Amongst Staff Caring for Intellectual Disability Patients in Long-Term Segregation: A Mixed Methods Study

**DOI:** 10.1192/bjo.2026.11620

**Published:** 2026-06-30

**Authors:** Gabriel Michael, Larteque Lawson

**Affiliations:** Bradford District Care NHS Foundation Trust, Bradford, United Kingdom

## Abstract

**Aims:**

This study aims to explore the prevalence and nature of moral injury amongst staff working with patients with intellectual disabilities in Long-Term Segregation (LTS) as well as identifying contributing factors as well as perceived support and coping strategies.

**Methods:**

A mixed methods design was utilised in a learning disability assessment and treatment unit (ATU) setting. This involved the collection of qualitative and quantitative data utilising anonymous surveys. Quantitative data being collected using a modified moral injury rating scale to explore degree of moral injury and wellbeing. Qualitative data was collected through exploration of staff experiences as well as any ethical dilemmas and emotional responses to LTS at work. For the quantitative data we employed the use of correlational analysis. For the quantitative data, thematic analysis was utilised.

**Results:**

The results do indicate that there may be moral injury that is experienced by staff involved in the care of intellectual disability patients in LTS. There appear to be certain factors that do contribute to this including prolonged use of LTS, perceived lack of flexibility in the system as well as limited opportunities for reflective practice.

**Conclusion:**

This piece of work clearly demonstrates the relevance of moral injury in staff working with individuals in LTS. It furthermore highlights the occupational risk for those involved as well as the importance of ensuring that this is addressed to note only prevent burnout, sickness absence and retention of staff but to chiefly ensure that standard of patient care are maintained. This work further highlights the need for more research into this area in order to improve the understanding of how moral injury develops and evolves over time.

